# Analyzing ecological restoration strategies for water and soil conservation

**DOI:** 10.1371/journal.pone.0192325

**Published:** 2018-02-09

**Authors:** Sandra Isay Saad, Jonathan Mota da Silva, Marx Leandro Naves Silva, João Luis Bittencourt Guimarães, Wilson Cabral Sousa Júnior, Ricardo de Oliveira Figueiredo, Humberto Ribeiro da Rocha

**Affiliations:** 1 Graduate Program of Environmental Science, Institute of Energy and Environment, University of São Paulo, São Paulo, Brazil; 2 Department of Atmospheric and Climatic Sciences, Federal University of Rio Grande do Norte, Natal, Brazil; 3 Departamento de Ciência do Solo, Universidade Federal de Lavras, Lavras, Brazil; 4 Aquaflora Meio Ambiente, Curitiba, Brazil; 5 Department of Water Resources and Environment, Aeronautics Institute of Technology, São Jose dos Campos, Brazil; 6 Embrapa Environment, Brazilian Agricultural Research Coorporation, Jaguariúna, Brazil; 7 Department of Atmospheric Science, Institute of Astronomy, Geophysics, and Atmospheric Sciences, University of São Paulo, São Paulo, Brazil; Oregon State University, UNITED STATES

## Abstract

The choice of areas for nature conservation involves the attempt to maximize the benefits, whether by carrying out an economic activity or by the provision of Ecosystem Services. Studies are needed to improve the understanding of the effect of the extent and position along the watershed of restored areas on soil and water conservation. This study aimed to understand how different restoration strategies might reflect in soil conservation and sediment retention. Using InVEST tool, sediment transport was simulated in a small 12 km^2^ watershed (Posses River, in Southeast Brazil), where one of first Brazilian Payment for Ecosystem Services (PES) projects is being carried out, comparing different hypothetical restoration strategies. With 25% of restoration, sediment export decreased by 78% for riparian restoration, and 27% for the steepest slopes restoration. On the other hand, the decrease in soil loss was lower for riparian restoration, with a 16% decrease, while the steepest slopes restoration reduced it by 21%. This mismatch between the reduction of sediment export and soil loss was explained by the fact that forest not only reduces soil loss locally but also traps sediment arriving from the upper parts of the watershed. While the first mechanism is important to provide soil stability, decreasing the risk of landslip, and to maintain agricultural productivity, the second can improve water quality and decrease the risk of silting, with positive effects on the water reservoirs at the outlet of the watershed. This suggests that Riparian and the Steepest Slopes restoration strategies are complementary in the sense of preventing sediments from reaching the water bodies as well as protecting them at their origin (with the reduction of erosion), so it will be advisable to consider the two types of restoration.

## Introduction

While the conservation of natural resources in not valued in traditional economics [1], there is a growing perception of the strong human dependence on Ecosystem Services (ES) [2]. Attempts to avoid ecosystem degradation face great challenges: on one hand, most of policies to avoid deforestation have been inefficient [3], and, on the other, ecosystem restoration is costly, and funds are generally limited [4]. Around the world, billions of dollars have been spent on ecosystem restoration programs which have not always been successful [5,6]. In fact, there is a need to choose priority zones for nature conservation that consider both environmental and social-economic issues as conservation priorities are needed for planning and decision making [7]. When it comes to achieving the economic viability of Payment for Ecosystem Services (PES) projects, a key factor is to choose potential areas for large production of ES [8] as targeted restoration can be much more efficient than random reforestation [9]. The cost of restoration can be used as a criterion for prioritization [4], and many times passive restoration can be employed to reduce costs due to the relatively good cost-effectiveness [6].

Besides fresh water springs, soil conservation projects often target two types of areas: riparian zones and steep slopes [10,11]. Riparian vegetation acts as a buffer which filters the sediments, nutrients and pollutants that may otherwise reach the streams; it helps to stabilize stream banks, increases flood control, and provides habitats for both aquatic and terrestrial species and acts as an ecological network, increasing habitats connectivity [12]. On the other hand, steep slopes are highly susceptible to landslip and contribute to the increase in erosion and sediment exportation in the watershed [7,13], and therefore vegetation is crucial to maintain the stability of slopes due to root water uptake and especially the reinforcement of soil structure by plant roots [14].

In Brazil, the Forest Act (Law #4.771/1965 and its revision in 2012, Law #12,651/2012) is a legal instrument to support ecosystem conservation on private agricultural land, with the protection of river banks, springs, steep slopes, and hilltops, which are called Permanent Preservation Areas (PPA), and, additionally, Legal Reserves (LR), which contain also other land features. In the current version of the Forest Act, some rules have been relaxed as they were not followed by most of the landowners, and if they were enforced by the government this could lead to huge economic losses. After many discussions between the members of congress, the 30 meters of river buffer (on both margins) remained in the 2012 revision, although its extend decreased [15]. For example, in the 1965 version it is counted from the longest bed, and in the 2012 version from the regular bed [16]. On the other hand, the PES schemes have emerged as a financial compensation to Brazilian rural landowners for the provision of Ecosystem Services and may encourage the enforcement of the Forest Act, which can help also with the restoration costs, a major obstacle for its compliance [17]. Like many other PES schemes around the world, in Brazil payment is generally based on opportunity costs and one common difficulty is the difficulty in monitoring the gain in Ecosystem Services, necessary in order to encourage its continuation and growth [18].

Despite the importance of ES measurements for decision making [19], there are still few studies which evaluate the influence of the spatial distribution of the natural areas providing ES under alternative scenarios of land use [9], including those within areas receiving interventions under PES projects [18]. Some studies have compared the efficiency in providing ES in specific pre-defined scenarios to prioritize some services like black bear and bird habitats, carbon storage, biodiversity, tourism, and others [6,9,12], but they did not explore how the efficiency changes with a gradual increase in the restoration area. Another issue for ES provision estimation under alternative land use scenarios is to account for the uncertainties and the need to inform decision makers on the limitations of the methodology employed [20], which few studies of model-based evaluation of land-use change effects have considered [21].

This study attempts to understand how different restoration strategies may reflect in the provision of two ES: the reduction of erosion and of sedimentation. We used an ensemble modeling approach, which considers the uncertainties in input data. The different strategies evaluated were restoration in riparian zones, restoration of steep slopes, a mix of the two, and that which was effectively implemented by the PES Project.

## Methodology

### 2.1 Study area

**Fig 1:**
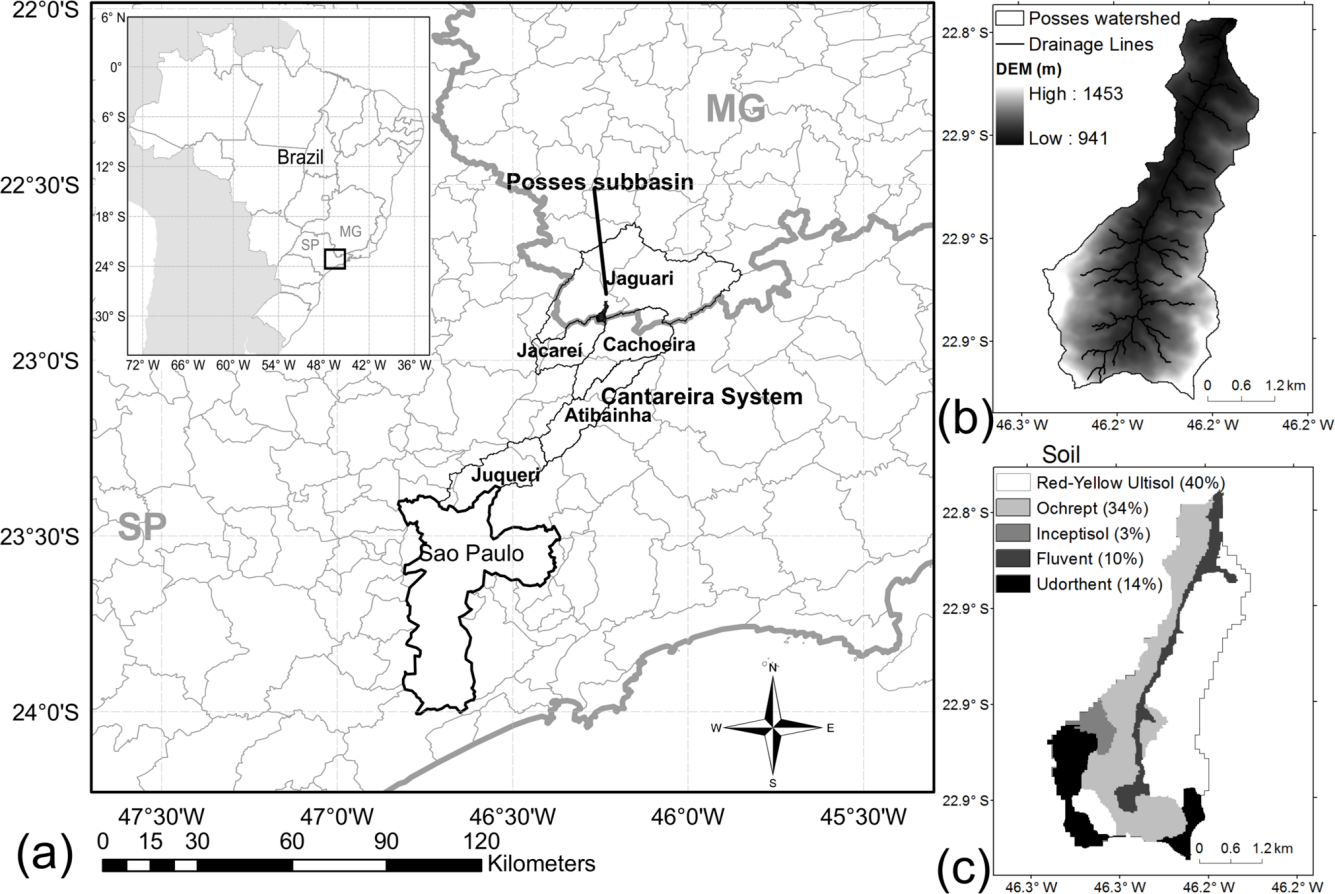
(a) Location of: Posses watershed, the municipality of São Paulo, and the main watersheds of Cantareira System, in the states of São Paulo (SP) and Minas Gerais (MG) in Brazil; (b) elevation (Digital Elevation Model, DEM, in m, with a pixel size of 30 m) in Posses watershed and drainage line; and (c) soil classes in the watershed.

**Fig 2:**
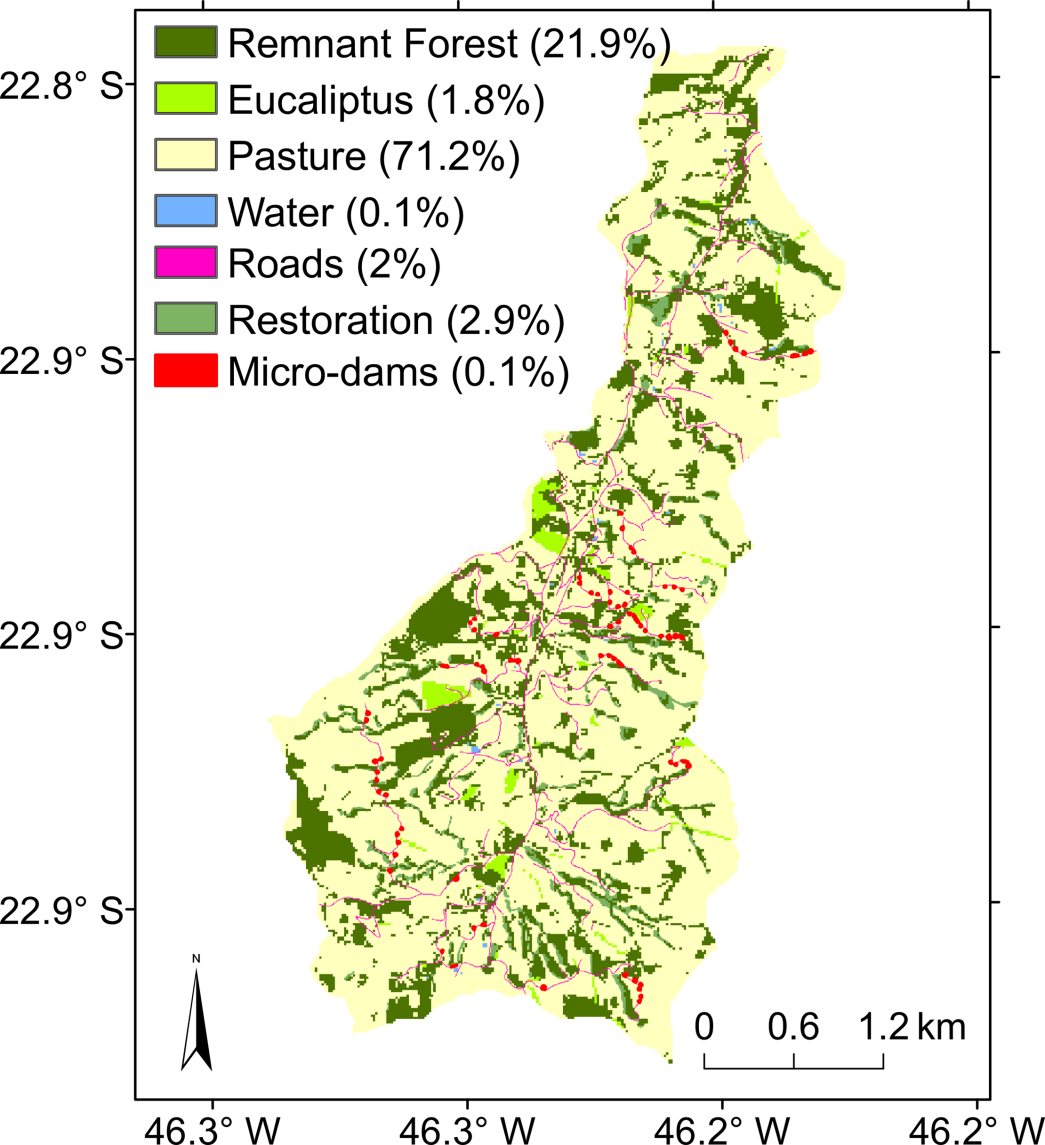
Current land-use in the Posses watershed. The area of the micro-dams was amplified for easy viewing. Source: [23].

Simulations were held for the Posses watershed (Fig 1), a sub-basin of the Jaguari watershed, which feeds part of the Cantareira Water supply system and provides almost half of the water for the megacity of São Paulo. Posses is located in the municipality of Extrema, in the State of Minas Gerais, where Conservador das Águas (Water Conserver), a pioneer PES project in Brazil, has been active since 2005 [17]. Posses is a rural watershed with an area of 1200 ha, inhabited by 53 small landowners. Due to its steep relief (Fig 1) and its rocky outcrops, extensive livestock is the predominant agricultural activity, with pastureland being the main land use in the watershed (71% of the area, Fig 2). With the PES project, most of the remaining original Atlantic forest (22% of the watershed) has been fenced off, and other areas of the watershed have been reserved for ecological restoration (Fig 2) with the potential to preserve and increase native forest cover to 25%. Other actions of the PES project have included the renovation of unpaved roads and the construction of micro-dams, known in Brazil as “*Barraginhas*”, which are designed nearby the roads to collect and store sediment and surface runoff [22].

Posses has a mean altitude of 1079 m, with mean monthly temperature varying between 14.5 and 21.5°C, an annual precipitation of around 1500 mm, with most precipitation occurring in the summer. The soils are generally shallow, with the following classes: Red-Yellow Ultisol, Ochrept, Inceptisol, Udorthent, and Fluvent. Red-Yellow Ultisol is predominantly composed of clay, while Inceptisol and Udorthent are mostly composed of sand. Udorthent is a shallower soil, with rocky outcrops, while Ultisol, Ochrept, and Inceptisol are relatively deep, but also with rocky characteristics. More information on the soils of the region can be found in [24].

### 2.2 Sediment export estimation

**Fig 3:**
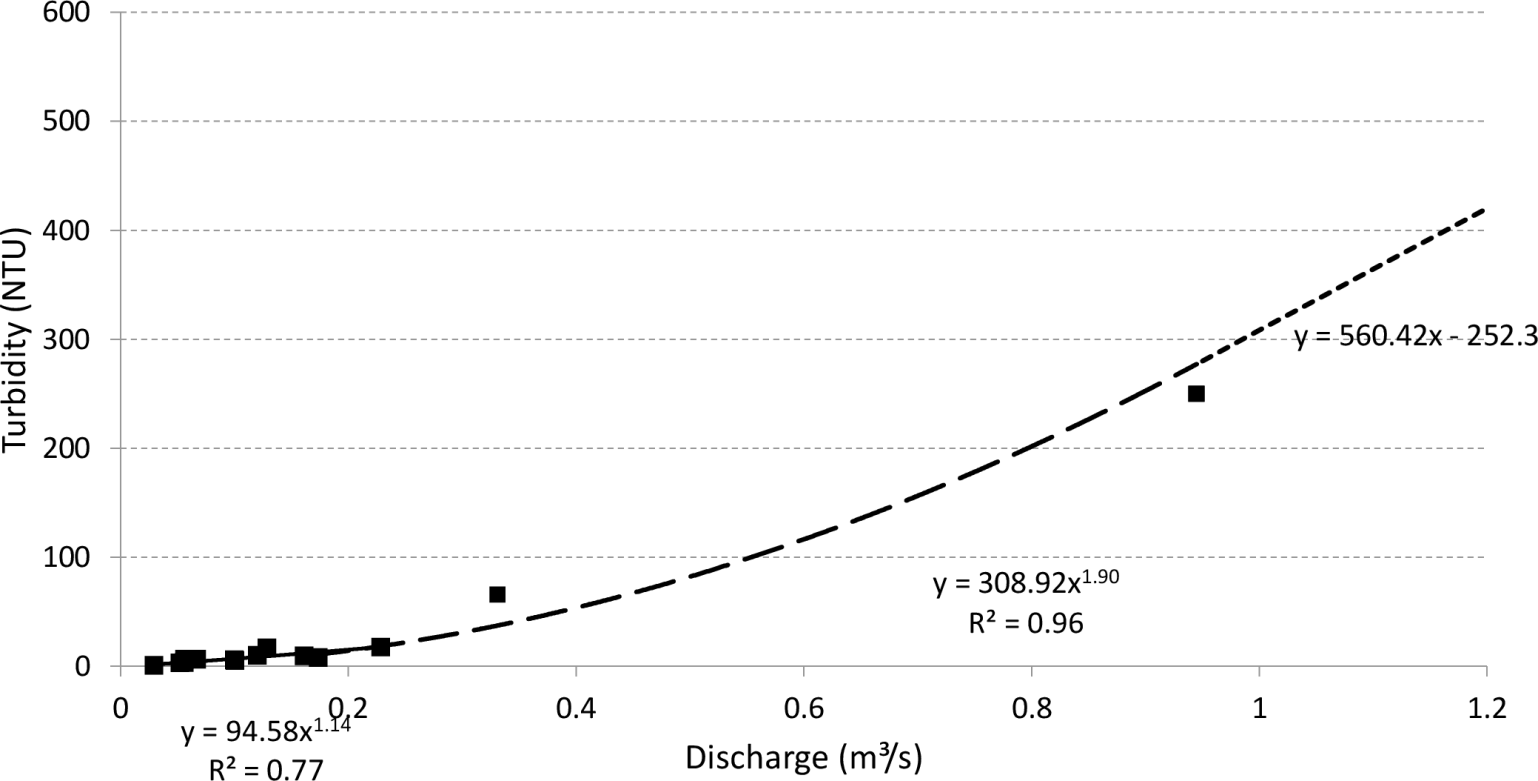
Turbidity as function of discharge for 18 samples in the mouth of Posses watershed.

In Posses watershed, the Brazilian Water Agency (*Agência Nacional das Águas*, ANA), with the support of the Extrema Municipality, installed a gauge in the outlet as well as five pluviometers scattered over the watershed for daily reading [25], which data are available at http://www.snirh.gov.br/hidroweb. In order to estimate sediment export from ANA observed data, twice a day water level data from October 2010 to September 2015 was converted into streamflow by flow rating curves estimated with available discharge measurements. Then, using some samples of turbidity and streamflow, we found a relation between turbidity and water discharge (Eq 1, Fig 3). Power ratio functions used to describe turbidity as function of discharge were also used by Strauch et al. [21], motivated by its traditional use for suspended-sediment rating curves [26].

$$TU[\mathrm{NTU}]=\left\{ \begin{array}{l} 94.58∙Q{\left[m^{3}s^{‑1}\right]}^{1.14}\;\mathrm{for}\;Q<0.23\;m^{3}s^{‑1}\\ 308.92∙Q{\left[m^{3}s^{‑1}\right]}^{1.90}\;\mathrm{for}\;0.23\;m^{3}s^{‑1}\leq Q<0.95\;m^{3}s^{‑1}\\ 560.42∙Q[m^{3}s^{‑1}]-252.3\;\mathrm{for}\;Q\geq 0.95\;m^{3}s^{‑1} \end{array}\right.$$ (Eq 1)

where TU is turbidity and Q is the discharge.

From turbidity, obtained using (Eq 1) (according the range of streamflow values), suspended-sediment concentration was calculated, using the relation of Lima et al. [27] (Eq 2), who made an estimate for another Brazilian watershed with similar characteristics.

$$SS[mgL^{-1}]=1.114∙TU[NTU]+1.4731$$ (Eq 2)

where SS is suspended-sediment concentration. Finally, annual sediment export values, divided by the area of the sub-basin, are given by:

$$SE[\mathrm{Mg}\;{\mathrm{ha}}^{‑1}y^{‑1}]=\frac{31.536∙Q[m^{3}s^{‑1}]∙SS[mg\;L^{-1}]}{DA[\mathrm{ha}]}$$ (Eq 3)

where SE is the sediment export, and DA is the drainage area of the watershed. The value of 31.536 is due to conversion of units.

**Fig 4:**
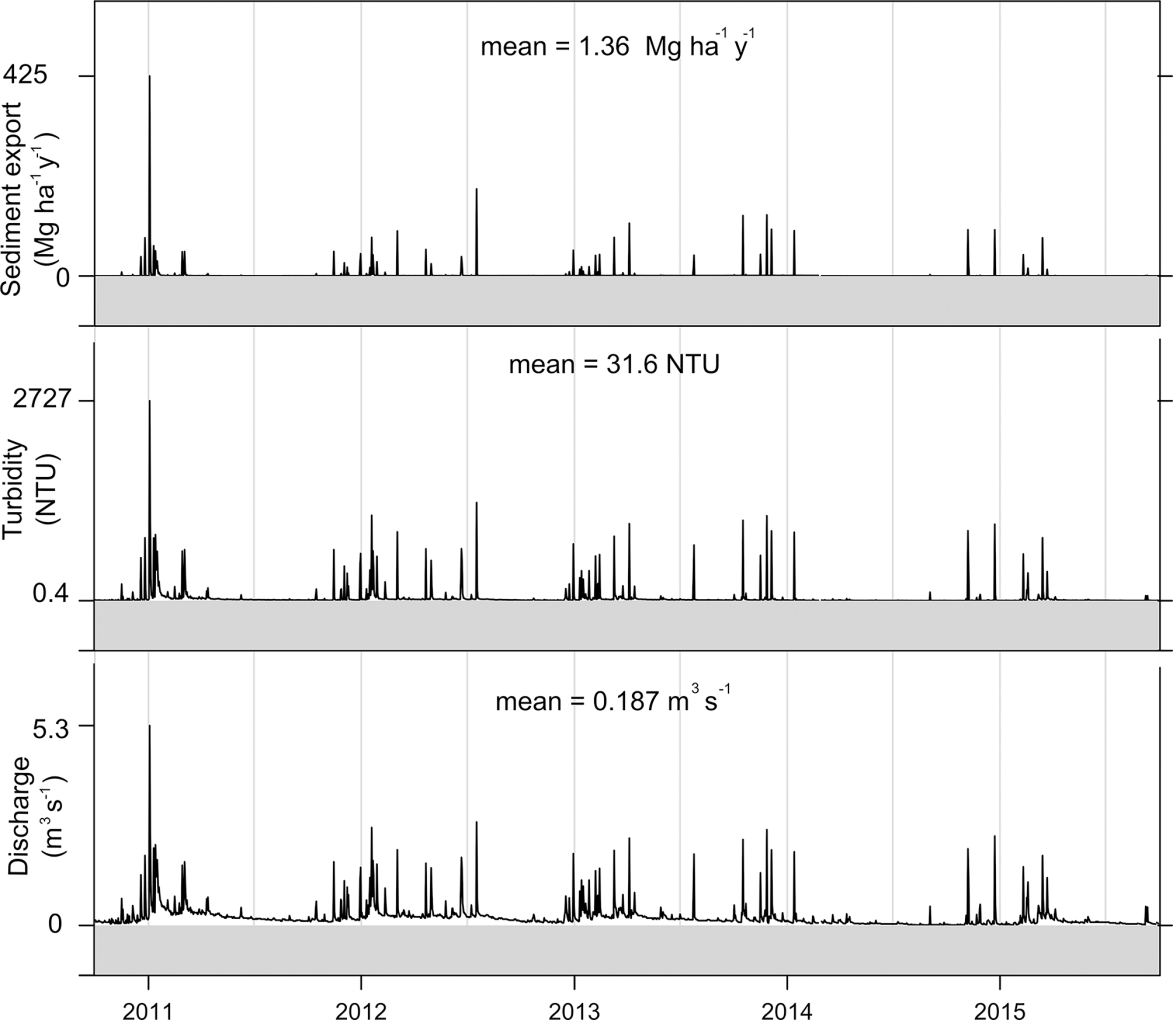
Temporal series of twice-a-day data of discharge (observed), turbidity (calculated with (Eq 1)) and sediment export (calculated with (Eq 3)) in the mouth of Posses Watershed, from October 2010 to September 2015. On the right, the percentage frequency distribution of the same variables.

The temporal series of discharge, turbidity, and sediment export in the mouth of Posses watershed are shown in Fig 4. The seasonality of discharge is noticeable by the variation in its peaks and low flows, both of which are greater in summer. The peaks are a consequence of local and intense showers that cause fast surface runoff due to the small size of the watershed and its steep relief. Moreover, turbidity and especially sediment export are highly sensitive to the discharge peaks. Annual values of discharge varied between 0.10 and 0.25 m^3^/s, turbidity between 15 and 42 NTU, and sediment export between 0.9 and 1.9 Mg ha^-1^ y^-1^. The mean turbidity (32 NTU) is lower than the maximum accepted value in raw water for treatment and human consumption (100 NTU), according Brazilian legislation [28], and below the level which hinders fish reproduction (50 NTU) [29]. The mean sediment export (1.36 Mg ha^-1^ y^-1^) is within the expected range of Brazilian rivers, between 0.03 and 1.70 Mg ha^-1^ y^-1^ [30]. In Pipiripau and Descoberto Lake Rivers Basins, in Central Brazil, with areas of 188 km^2^ and 105 km^2^ respectively, the estimate ranges from 0.10 to 0.26 Mg ha^-1^ y^-1^ [21]. The estimate for Posses watershed is higher than these because it is a smaller watershed (approximately 10 times lower), and there is a scaling effect with higher export in smaller basins [31].

### 2.3 InVEST model

Simulations were performed with the “Avoided Reservoir Sedimentation Model” of InVEST (Integrated Valuation of Ecosystem Services and Tradeoffs, version 2.4), a set of models developed to map and evaluate the Ecosystem Services (ES) [32]. InVEST includes the modeling of the sedimentation biophysical processes and the valuation of sediment reduction ES. This study only used the biophysical module, which aims to model sediment transport from the erosion areas to the point where sediments are trapped or reach a water body. Soil loss is calculated in each pixel, using the Universal Soil Loss Equation (USLE, (Eq 4)) [33], which has been used worldwide, including in Brazil sites, as it requires relatively little input data and adaptations in parameterizations [34].

A=R∙K∙LS∙C∙P (Eq 4)

where *A* is the soil loss (Mg ha^-1^y^-1^), *R* is the rainfall erosivity (MJ mm ha^-1^ h^-1^y^-1^), *K* is the soil erodibility factor (Mg h ha MJ^-1^mm^-1^ha^-1^), LS is the slope length-gradient factor, *C* is the crop/vegetation and management factor, and *P* is the support practice factor.

**Fig 5:**
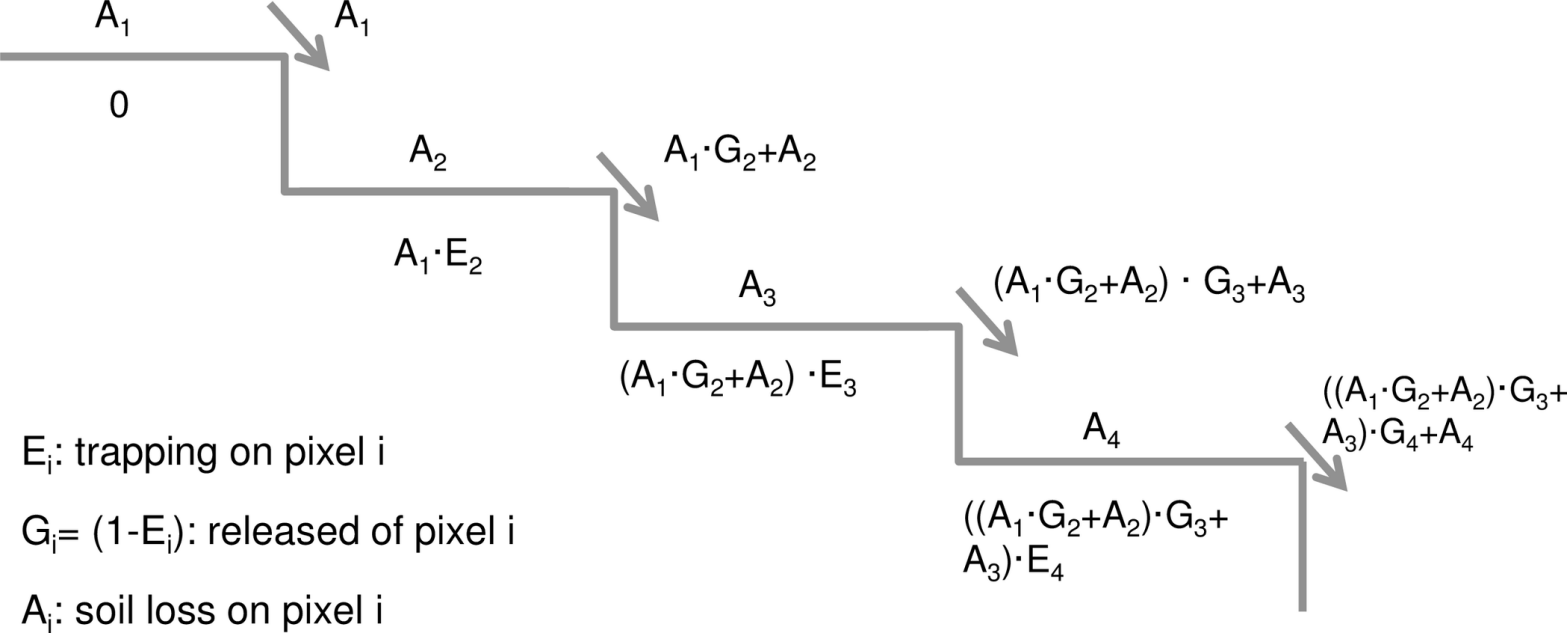
Simplified illustration of sediment route in InVEST model.

The soil eroded in each pixel is routed downstream along a flowpath, and part of it is trapped by downstream vegetation, which depends on the sediment retention efficiency parameter for each land-use. The outflow quantity of sediment from cell *n* (*O*_*n*_) is given by:

$$O_{n}=A_{1}G_{2}G_{3}G_{4}\ldots G_{n}+A_{2}G_{3}G_{4}\ldots G_{n}+A_{3}G_{4}\ldots G_{n}+A_{4}\ldots G_{n}+\ldots +A_{n}$$ (Eq 5)

where *A*_*n*_ is the soil loss on pixel n *(Mg)*, and *G*_*n*_ (one-dimensional) is the fraction of the sediments which are not trapped in the way towards a water body, and is given by:

$$G_{i}=(1-E_{i})$$ (Eq 6)

where *E* (one-dimensional) is sediment retention efficiency of vegetation on the pixel *i*. It is a parameter that must be set in InVEST for each land use. This process is shown in a simplified illustration in Fig 5 in only two dimensions (one horizontal dimension is suppressed, for simplicity).

The outputs of InVEST model used in this study were: soil loss map, which corresponds to *A* in (Eq 4), and its mean in the watershed; upstream sediment retention, which is the fraction of sediment retained from sediment originating upstream in each pixel; sediment export in the outlet, which is *O*_*n*_ divided by the area of the watershed; and sediment export map, which quantifies sediments arriving in each pixel.

#### 2.3.1 Model setup

**Fig 6:**
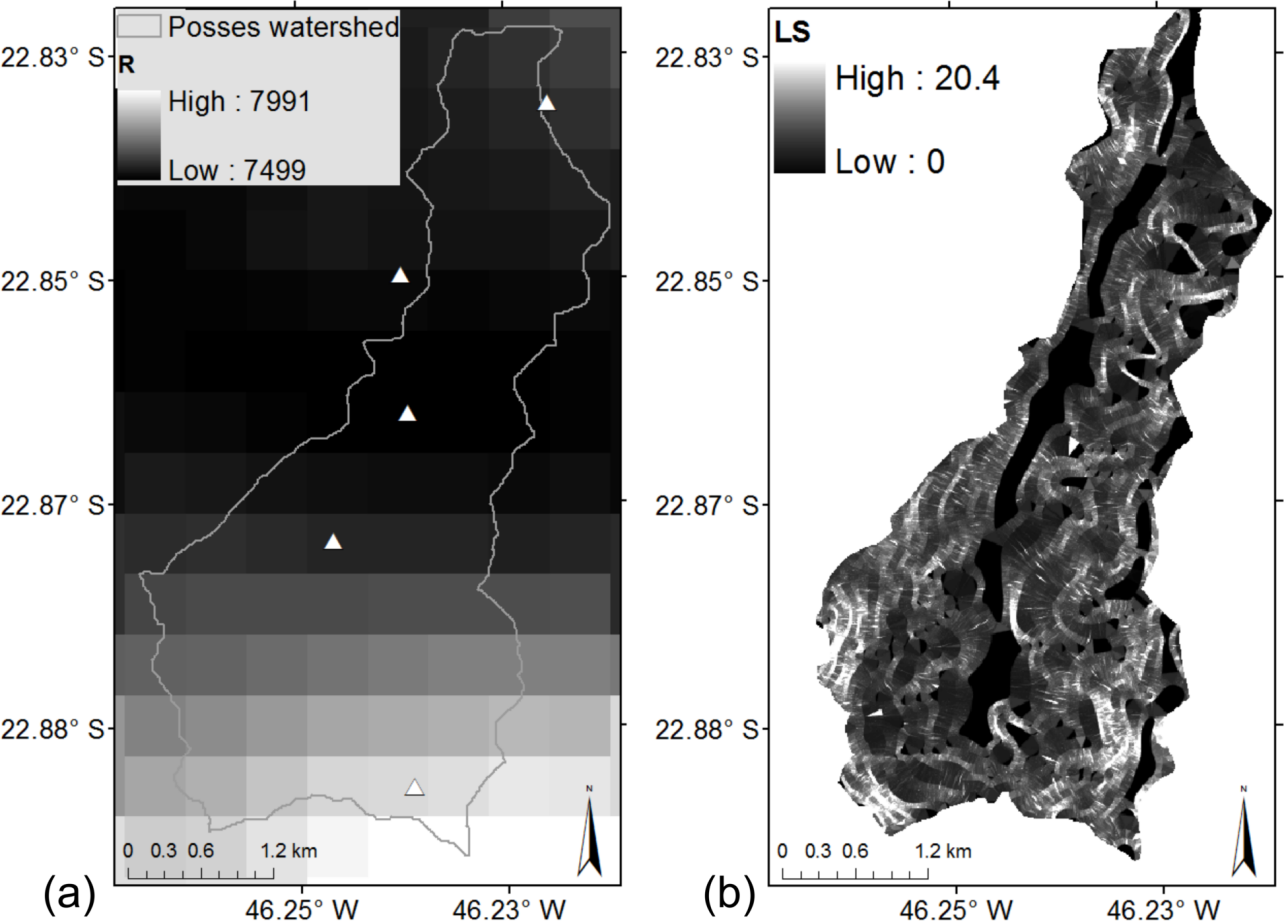
(a) Rainfall erosivity, in MJ mm ha^-1^h^-1^y^-1^, calculated from monthly precipitation data from five pluviometers spread out along the Posses watershed (white triangles), using data from Oct 2010 to Sep 2015. (b) Slope length-gradient factor (LS) input in InVEST Model, obtained from Zolin et al. [37].

Rainfall erosivity was calculated using daily precipitation data from October 2010 to September 2015 from five pluviometers along the Posses watershed, provided by ANA. From the daily precipitation data, monthly total precipitation was computed and interpolated spatially using the Cressman method [35], and finally calculated rainfall erosivity (Fig 6), using Lombardi Neto and Moldenahuer [36] relationship (Eq 7), whose results were similar to those from the original USLE method [33].

$$R=68,730\sum_{t=1}^{12}{\left({p_{t}}^{2}/P\right)}^{0,841}$$ (Eq 7)

where R is rainfall erosivity, in MJ mm ha^-1^h^-1^y^-1^, p_t_ is the monthly rainfall, in mm, and P is annual rainfall, in mm.

An observational study in the studied region found a large variation in soil erodibility for the same soil classes [38]. Minimum, mean, and maximum values of *K* calculated for each soil class in that study (Table 1) were considered as possibilities for the calibration step.

**Table 1:** Minimum, maximum, and mean erodibility factor for the soils of Posses watershed. Data was obtained from Avalos (38).

Soil class	Minimum	Maximum	Mean
	(t h MJ^−1^ mm^−1^)		
Red-Yellow Ultisol	0.0139	0.0386	0.0252
Ochrept	0.0093	0.0398	0.0245
Inceptisol	0.0105	0.038	0.0225
Udorthent	0.0137	0.0326	0.0233
Fluvent	0.008	0.0377	0.0236

The slope length-gradient factor (LS) was obtained from Zolin et al. [37] (Fig 6B), who used the Desmet and Govers [39] algorithm. Higher values of LS occurred on the higher slopes (not shown).

For the three major land uses, pasture, forest, and eucalyptus plantations, we have defined an initial range for the *C* factor (Table 2), according to Martins et al. and Silva et al. [34,40,41]. The final *C* factor for these land uses was obtained by calibration of sediment export. For the unpaved roads, water and micro-dams, the values of C prescribed were respectively, 1, 0, and 0, and for the growing forest (around 5 years after they were planted), the mean of the calibrated C values for pasture and forest.

**Table 2:** Possible minimum and maximum values of *C* factor of pasture, forest, and eucalyptus considered in calibration.

	C factor		
	Pasture	Forest	Eucalyptus
Minimum	0.05	0.02	0.12
Maximum	0.22	0.09	0.3

Two different methodologies were employed to estimate Sediment Retention Efficiency (SRE) for pasture and forest. As pasture is the predominant land use in the watershed in the current (control) scenario, used for model calibration, the parameter was obtained through calibration, as described later in the *Calibration method* Section. With regard to the forest, which is expected to act as a sediment buffer, its SRE was chosen by a literature search [42–44]. As these references refer to the Trapping Efficiency which is different from the Invest SRE parameter, we built a relation between them:

Trapping Efficiency is defined as the ratio between the total mass flowing onto and out of the buffer zone [44] (Eq 8).

$$T_{e}=\left(M_{i}-M_{o}\right)/M_{i}$$ (Eq 8)

where T_e_ is trapping efficiency, M_i_/M_o_ is total mass flowing onto/out of the buffer zone (in Mg ha^-1^).

For a homogeneous vegetation buffer (with homogeneous SRE), and considering soil loss inside it is null (i.e. *A*_2_ = *A*_3_ = *A*_4_ = … = *A_n_* = 0), (Eq 5) can be rewritten as:

$$O_{n}=A_{1}G_{2}G_{3}G_{4}\ldots G_{n}=A_{1}{G_{n}}^{n}=A_{1}{\left(1-SRE\right)}^{n}$$ (Eq 9)

and (Eq 8) gives:

$$T_{e}=\left(A_{1}-O_{n}\right)/A_{1}=1-{\left(1-SRE\right)}^{n}$$ (Eq 10)

The number of pixels in the buffer (n) is its width (*l*) divided by the pixel size (r). So, (Eq 10) gives:

$$T_{e}=1-{\left(1-SRE\right)}^{l/r}$$ (Eq 11)

**Fig 7:**
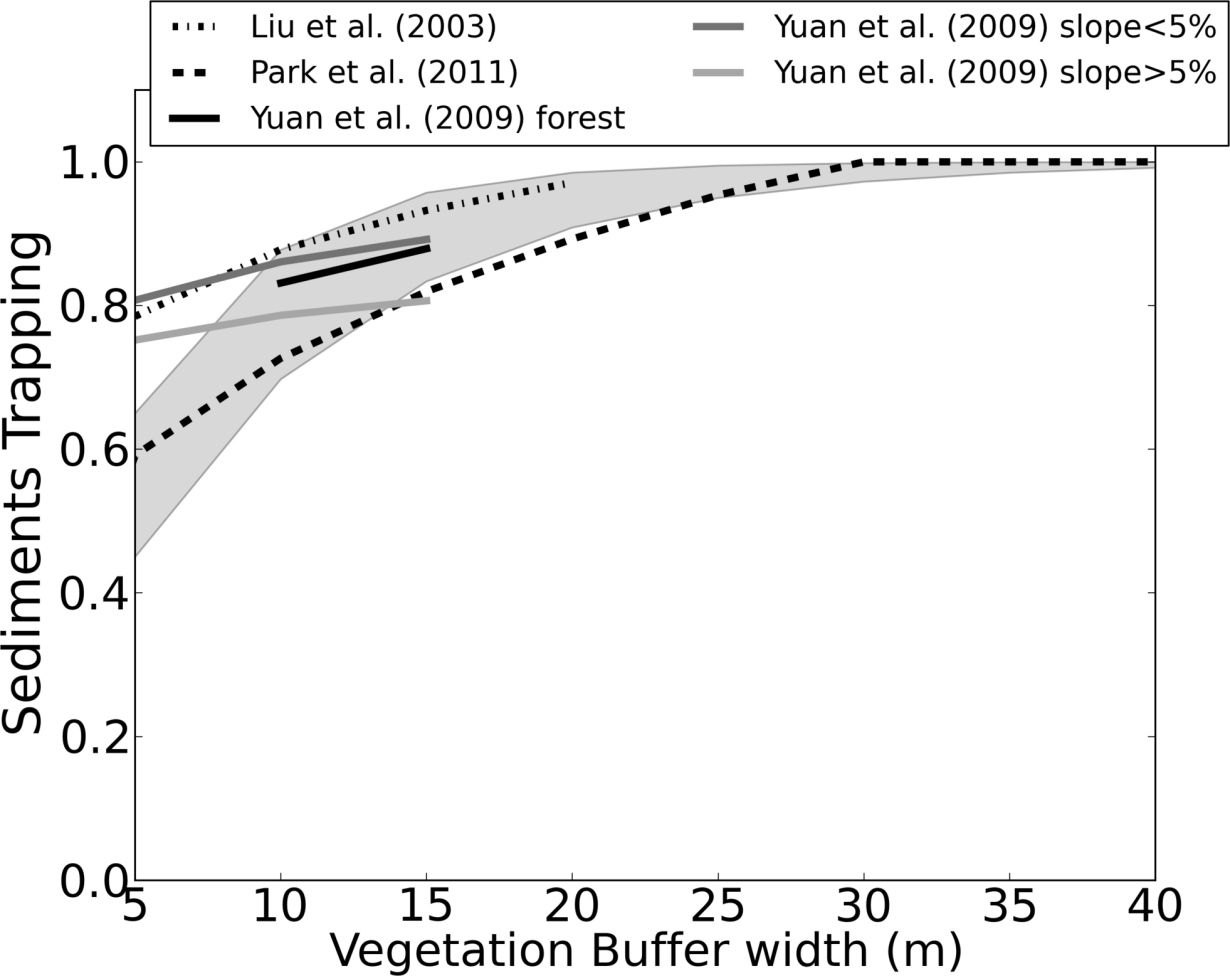
Relationship between sediments trapping and vegetation buffer width suggested by Liu et al., Park et al., and Yuan et al. [42–44], and for the Sediment Retention Efficiency of InVEST between 45% and 65% (in shaded).

Therefore, trapping efficiency was calculated as function of InVEST SRE and buffer width *(l*). Fig 7 shows the Trapping from references [42–44] as function of vegetation buffer width, and also the Trapping calculated using SRE equals to 45% and 65%. This threshold of SRE between 45–65% was the one that best fitted the references’ Trapping (shaded in Fig 7), and it was the chosen interval to prescribe forest SRE.

Table 3 lists InVEST input data and parameters. For some parameters, we used more than one value: the *K* factor, as it is one of the most sensitive parameters for sediment export modeling in InVEST [45] and due to the variations previously mentioned; the *C* factor, for which literature values differ widely [34,40,41]; Sediment Retention Efficiency, due to the lack of observed data or regional references. We fixed the *R* factor, due to the inexistence of other data with comparable quality and resolution; the LS factor, as the chosen method is the most widely used; and threshold flow accumulation, whose sensitivity was low (not shown). Spatial resolution used in the simulation was of 5 m.

**Table 3:** Parameters and input data used in the model.

Input	Type	Data	Source/Calculation
DEM	Raster	Fig 1	Advanced Spaceborne Thermal Emission and Reflection Radiometer (ASTER) with 30 m, and interpolated to 5 m.
*R* Factor	*Raster*	Fig 6A	Calculated using A.N.A’s precipitation data from Oct 2010 to Sept 2015
*K* Factor	*Raster*	Three options for each soil class, as shown Table 1	[38]
*C* Factor	Per land use	As shown in Table 2	Calibration considering the thresholds in the table.
*LS* Factor	*Raster*	As shown in Fig 6B	[37]
Sediment Retention Efficiency	Per land use	Forest: 45% and 65% Pasture: from 5% to 40% Eucaliptus: from 25% to 52% Micro-dams: 100% Others: 0%	Calculated comparing other sources Calibration Mean between forest and pasture
Threshold flow accumulation	Constant	2000	Chosen by sensibility tests comparing simulated to observed river network

#### 2.3.2 Calibration method

Sediment export was calculated from observational data (from now on, called as observed sediment export) and was used for model calibration. The mean value for the period from Oct 2010 to Sep 2015 was considered for model calibration and verification. The calibration process led the model by changing its parameters so that the simulated sediment export was as close as possible to the observed sediment export. The maximum error threshold considered for calibration was 10%. Calibration was performed by trial and error by changing one parameter at a time, so that while one parameter had its value changed until the modeling run estimates fell within the acceptable error limit, the other parameters remained constant.

Calibration considered uncertainties of model inputs, i.e., it applied more than one value option for parameters related to soil and land use cover (Table 4). Each member combined one *K* factor option (minimum, mean, and maximum, of Table 1), one option of the forest Sediment Retention Efficiency (45% or 65%), a minimum and maximum pasture *C* factor, and two options of variations of forest *C* with pasture *C*: one that forest (and also Eucaliptus) *C* increases, and one that it decreases linearly with pasture *C* (according to Table 2). For the cases where the minimum error condition was not achieved, the *C* factor was adjusted within the minimum and maximum of the initial range. And if the condition was still not reached, the member was disregarded.

**Table 4:** Specification of the members for calibration. Each member used two options of variations of forest *C* with pasture *C* (increases or decreases), a minimum, mean, or maximum *K* factor (Table 1), one of two possible values of Forest Sediment Retention Efficiency (45% and 65%), and an initial pasture *C* factor for calibration. Pasture Sediment Retention Efficiency was obtained through calibration considering a range between 5% and 40%.

	Calibration Member	K factor	Forest Sed. Retention Ef.	Initial C pasture factor for calibration	Pasture Sed. Retention Ef.
Forest C increases with pasture C	1a	minimum	45%	minimum	5%-40%
	2a	minimum	65%	minimum	5%-40%
	3a	minimum	45%	maximum	5%-40%
	4a	minimum	65%	maximum	5%-40%
	5a	mean	45%	minimum	5%-40%
	6a	mean	65%	minimum	5%-40%
	7a	mean	45%	maximum	5%-40%
	8a	mean	65%	maximum	5%-40%
	9a	maximum	45%	minimum	5%-40%
	10a	maximum	65%	minimum	5%-40%
	11a	maximum	45%	maximum	5%-40%
	12a	maximum	65%	maximum	5%-40%
Forest C decreases with pasture C	1b	minimum	45%	minimum	5%-40%
	2b	minimum	65%	minimum	5%-40%
	3b	minimum	45%	maximum	5%-40%
	4b	minimum	65%	maximum	5%-40%
	5b	mean	45%	minimum	5%-40%
	6b	mean	65%	minimum	5%-40%
	7b	mean	45%	maximum	5%-40%
	8b	mean	65%	maximum	5%-40%
	9b	maximum	45%	minimum	5%-40%
	10b	maximum	65%	minimum	5%-40%
	11b	maximum	45%	maximum	5%-40%
	12b	maximum	65%	maximum	5%-40%

This ensemble modeling approach considering the uncertainties in the input data was shown to be more reliable than single model predictions [46,47], and the method can also be used to estimate the uncertainty of the predictions of land use change scenarios based on the dispersion of simulated results [21], which helps to appropriately inform decision makers [20].

### 2.4. Land use scenarios

**Fig 8:**
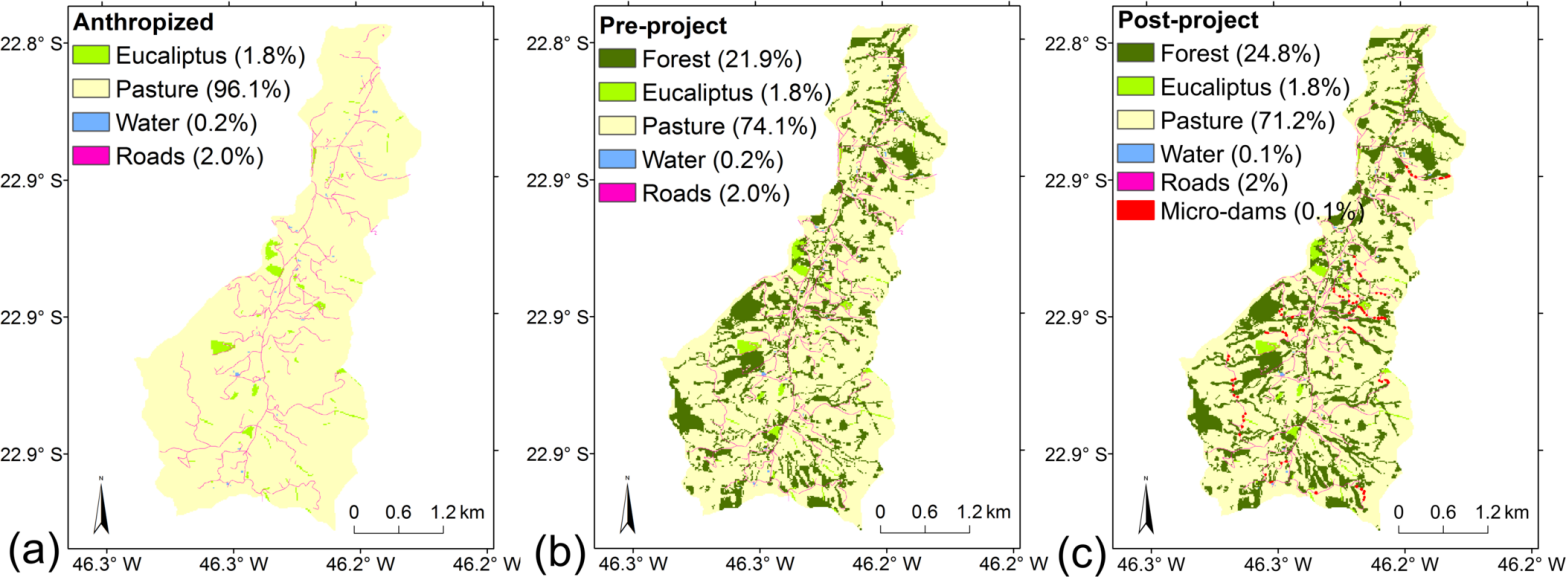
Simulated land use scenarios considered in the current study: (a) Anthropized, with no management and all remnant forest converted into pastureland; (b) Pre-Project, characterizing land-use at the beginning of Conservador das Águas Project in 2005; and (c) Post-Project, the land-use that may be achieved after some years from project start. In (c), the area of the micro-dams was amplified for easy viewing.

After calibration, which was based on the current land-use map (Fig 2), other land-use scenarios were developed in order to investigate the effect of different restoration strategies on sediment transport (Table 5). All calibrated members were considered in the scenarios. We used two scenarios, one before the beginning of the Conservador das Águas Project in 2005, and one a number of years after the start of the project, when the native species in restoration sites were completely developed. These scenarios were called Pre-Project (Fig 8B) and Post-Project (Fig 8C), respectively. We used an additional scenario that considered that no conservation practices had been carried out, with all remnant forest converted into pastureland, and this was called the Anthropized Scenario (Fig 8A). This strategy of increasing forest area from Anthropized Scenario to Pre-Project and to Post-Project was called the Conservador das Águas Project strategy.

**Table 5:** Summary of the scenarios used and restoration strategies and their characteristics.

Scenario/ Strategy	Characteristic	Used for	Land use fracions
Anthropized	No micro-dams and no remnant forest.	Evaluate changes in the ES of the Post-Project scenario in relation to this.The ES evaluated are due to Conservador das Águas Project land use change and current land use.	Pasture 96%; Roads: 2%; Eucaliptus: 2%
Pre-Project	Land-use before the beginning of Conservador das Águas Project: with no micro-dams; with remnant forest.	Evaluate changes in the ES of the Post-Project scenario in relation to this. The ES evaluated are due to Conservador das Águas Project land use change.	Pasture 74%; Native forest 22%; Roads: 2%; Eucaliptus: 2%
Current (Control)	Land-use during the period with observational data (2010–2015): with micro-dams; with remnant forest and in the beginning of restoration.	Model calibration	Pasture 71%; Native Forests 25% (22% of remnant and 3% in the beginning of restoration); Roads: 2%; Eucaliptus: 2%; Micro-dams 0.1%
Post-Project	Land-use achieved after Conservador Project: with micro-dams; with the remnant forest; and with the restoration (species completely recovered).	Evaluate changes in the ES due to the restoration actions of the PES.	Pasture 71%; Native Forests 25%; Roads: 2%; Eucaliptus: 2%; Micro-dams 0.1%
Riparian restoration strategy	Group of scenarios, starting from Anthropized Scenario and increasing forests along the river banks.	Evaluate changes in ES with the riparian restoration.	Forest: from 0% to 46%
Steepest Slopes restoration strategy	Group of scenarios, starting from Anthropized Scenario and increasing forests in the steepest slopes.	Evaluate changes in ES with the steepest slopes restoration.	Forest: from 0% to 45%
2-way restoration strategy	Group of scenarios, starting from Anthropized Scenario and increasing forests simultaneously in the steepest slopes and along the river banks.	Evaluate changes in ES with the riparian and the steepest slopes restoration.	Forest: from 0% to 49%
Conservador Strategy	Group gathering the scenarios Anthropized, Pre-Project, and Post-Project.	Evaluate changes promoted by Conservador das Águas Project.	Forest: 0% (Anthropized), 22% (Pre-project), and 25% (Post-Project).

**Fig 9:**
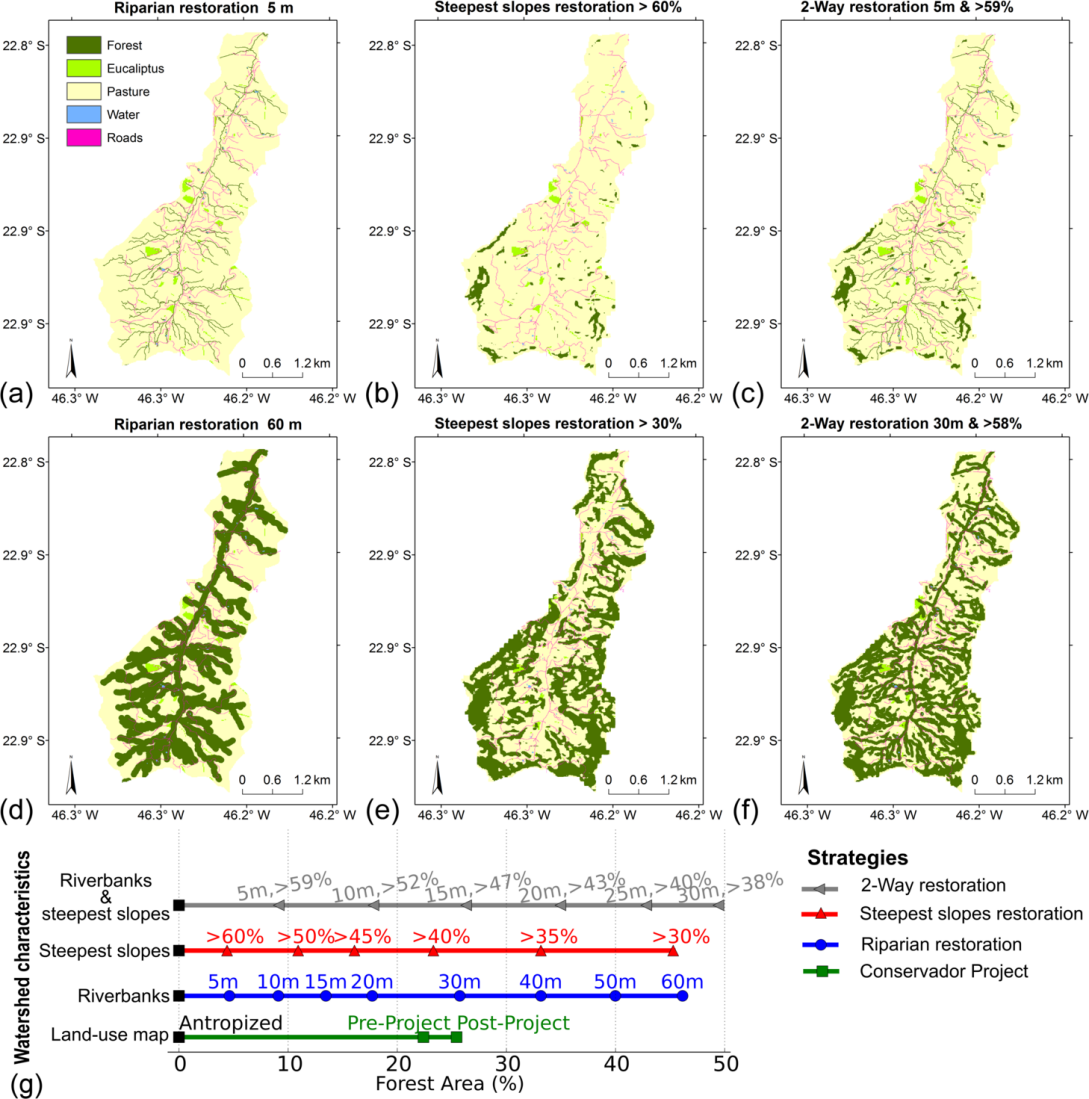
Simulated land use scenarios using different restoration strategies: (left) Riparian, (center) Steepest Slopes, and (right) in the two previous ways (2-Way). Other intermediate scenarios appeared in (g), which shows land-use scenarios as a function of the forest area of the watershed, for each restoration strategy: Conservador Project (in green), Riparian (blue), Steepest Slopes (red), and in the two previous ways (2-Way, gray).

Another three restoration scenarios were simulated, all of them originating from the Anthropized Scenario, with 0% of forest, and gradually increasing forest coverage until reaching approximately 45% of the area of the watershed, using different restoration strategies: along the riverbanks (Riparian restoration), on the steepest slopes (Steepest Slopes restoration), and simultaneously along the riverbanks and on the steepest slopes (2-Way restoration) (Table 5). The Riparian restoration strategy considered the gradual restoration from 5 to 60 m along the riverbanks (Fig 9A, 9D and 9G), and the reforestation of the steepest slopes considered the gradual reforestation starting from slopes with angles above 60%, gradually increasing the area so that it covers the entire area with a slope above 30% (Fig 9B, 9E and 9G). The 2-Way strategy gradually increased the area of forest using in each scenario the same proportion for riparian and for the steepest slopes reforestation (Fig 9C, 9F and 9G).

The comparison between the restoration strategies and scenarios was performed in two ways:

- Illustrating the model outputs, such as soil loss and sediment export, as function of the length of the restoration area, for each restoration strategy (Riparian, Steepest Slopes, 2-Way, and Conservador). It was used mainly for a qualitative analysis, to explore the difference between the strategies, and identify the most efficient ones;
- Choosing one scenario of each strategy with the same restoration area for quantitative analyses as well as exploring the spatial pattern of the model outputs in the watershed. Two cases were evaluated: (i) riparian restoration of 20 m, with an area of 9% of forest, and the others with a similar area (steepest slopes restoration > 43% slope and 2-Way reforestation of 10 m width & > 52% slope); (ii) Conservador Project strategy, which includes the Anthropized (0% of forests), Pre-Project (22%), and Post-Project (25%) scenarios, and those from the other strategies with the same area of forest (found by Fig 9G).

## Results

### 3.1 Sediment export calibration

The values for pasture and forest calibrated *C* and pasture Sediment Retention Efficiency for each calibrated member are shown in Table 6, with its estimated mean soil loss and sediment export. Each member considered *C* value variations within an established range (summed up in Table 4). The increase in pasture (the dominant land use in the watershed) *C* and pasture Sediment Retention Efficiency led to an increase and a decrease in sediment export, respectively (Table 6). Some members could not reach a desirable calibration (i.e., errors were greater than 10%) and were dismissed, which is the case of Members 1a-4a and 1b-4b, which used the lowest possible values of *K* factor. In these cases, simulated sediment export was very low in comparison to the observation, even using the maximum threshold of *C* and the minimum threshold of pasture Sediment Retention Efficiency.

**Table 6:** The calibrated values of pasture *C* and Sediment Retention Efficiency, and results of simulated soil loss and sediment export are listed for each calibrated member. In the empty cells, calibration was not successful, and its members were not considered. Mean absolute error from simulated sediment export was calculated using the observed sediment export (of 1.35 Mg ha^-1^y^-1^).

Calibration Member	Calibrated Pasture/ Forest C			Calibrated Pasture Sed. Retention Ef.	Soil Loss (Mg ha^-1^ y^-1^)	Sediment Export (Mg ha^-1^ y^-1^)	Absolute error (%) from the observed of 1.356 Mg ha^-1^ y^-1^
1a							
2a							
3a							
4a							
5a	0.200	/	0.082	5%	13.2	1.36	0.1%
6a	0.220	/	0.090	5%	14.4	1.33	1.8%
7a	0.220	/	0.090	6%	14.4	1.35	0.7%
8a	0.220	/	0.090	5%	14.4	1.33	1.8%
9a	0.118	/	0.048	5%	12.8	1.35	0.2%
10a	0.135	/	0.055	5%	14.4	1.35	0.1%
11a	0.220	/	0.090	13%	22.3	1.36	0.2%
12a	0.220	/	0.090	11%	22.3	1.35	0.4%
1b							
2b							
3b							
4b							
5b	0.215	/	0.022	5%	13.0	1.36	0.1%
6b	0.220	/	0.020	5%	13.2	1.23	9.0%
7b	0.220	/	0.020	6%	13.2	1.26	7.1%
8b	0.220	/	0.020	5%	13.2	1.23	9.0%
9b	0.113	/	0.064	5%	12.9	1.36	0.2%
10b	0.134	/	0.055	5%	14.3	1.36	0.1%
11b	0.220	/	0.020	12%	20.4	1.33	1.7%
12b	0.220	/	0.020	10%	20.4	1.33	1.6%
Mean					15.5	1.33	
Standard Deviation					3.9	0.01 Mg ha^-1^ y^-1^ (0.7%)	
Mean Abs Error					-	0.01	

Members 5a-12a and 5b-12b, and their average were successful in the calibration of the observed mean sediment export, with a mean absolute error of less than 1%. Variations between simulated sediment export of the calibrated members were very low (Table 6), with a standard deviation of 0.01 Mg km^-2^y^-1^. However, as calibration could not be performed for soil loss estimates (due to the inexistence of soil loss observational measures), the variability between the members was high, with a minimum of 12.8 Mg ha^-1^y^-1^, a maximum of 22.3 Mg ha^-1^y^-1^, and a standard deviation of 3.9 Mg ha^-1^y^-1^ (Table 6). Although no observational measures are available for soil loss validation, sediment yield is generally about one order of magnitude lower than soil erosion rates on hill slope plots [48], which is consistent with our results.

**Fig 10:**
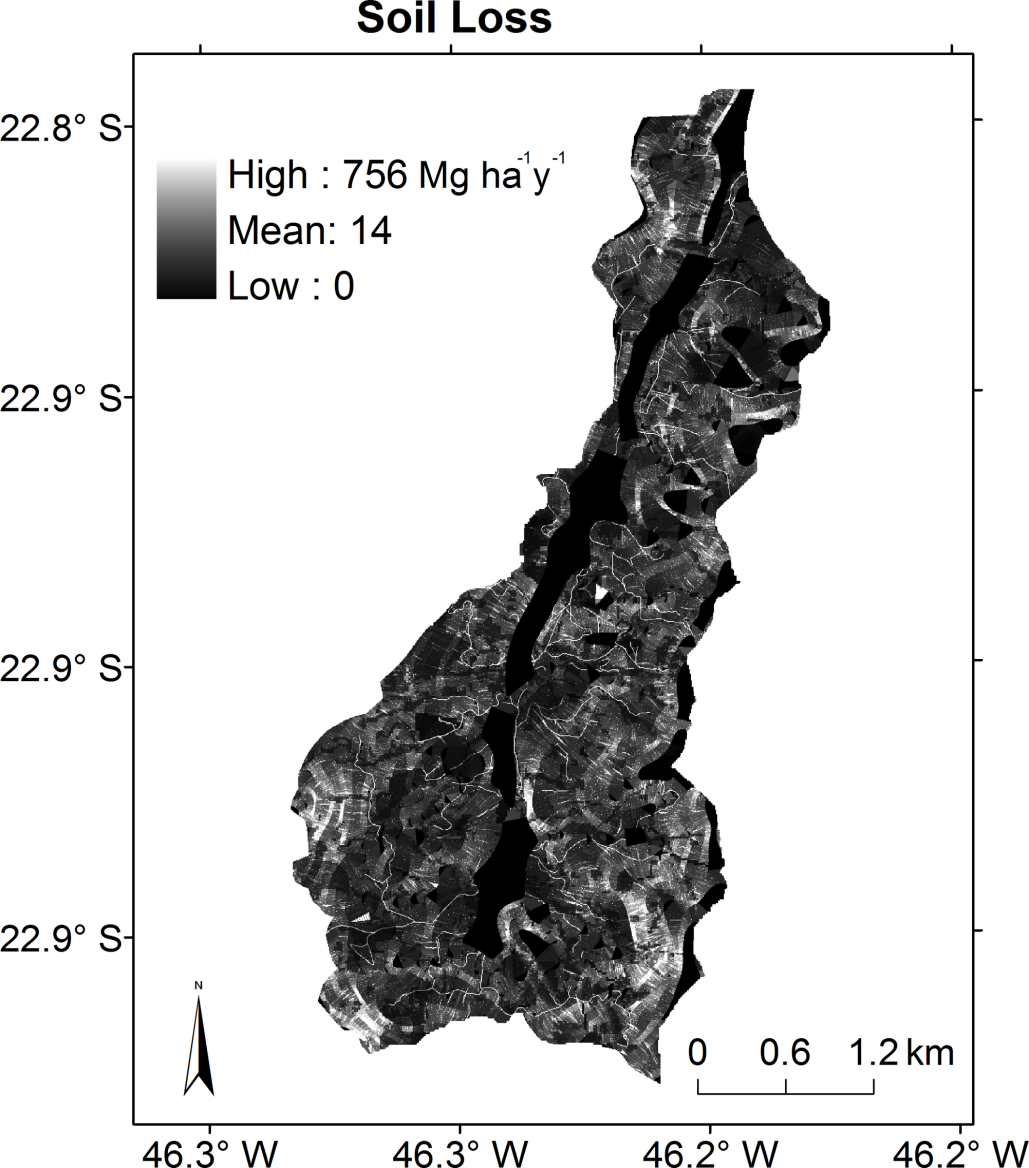
Simulated soil loss by calibration member 7a, in Mg ha^-1^y^-1^.

The highest values of soil loss were reached by members 11a and 12a (Table 6), with the highest values for *K* and pasture and forest *C* (Table 4), as the increase in each factor led to an increase in soil loss, compensated in the modelling process by a higher sediment retention parameter, with influence only on sediment export and not soil loss. The spatial pattern of soil loss was similar for all members despite the difference in magnitude (not shown). In general, the LS factor showed a greater control, for which maximum values of LS (Fig 6B) resulted in greater values of soil loss (Fig 10). Roads (Fig 2) also played an important role as erosion factors, as they resulted in a greater soil loss (Fig 10).

### 3.2 Effect of restoration on soil loss and sediment export

**Fig 11:**
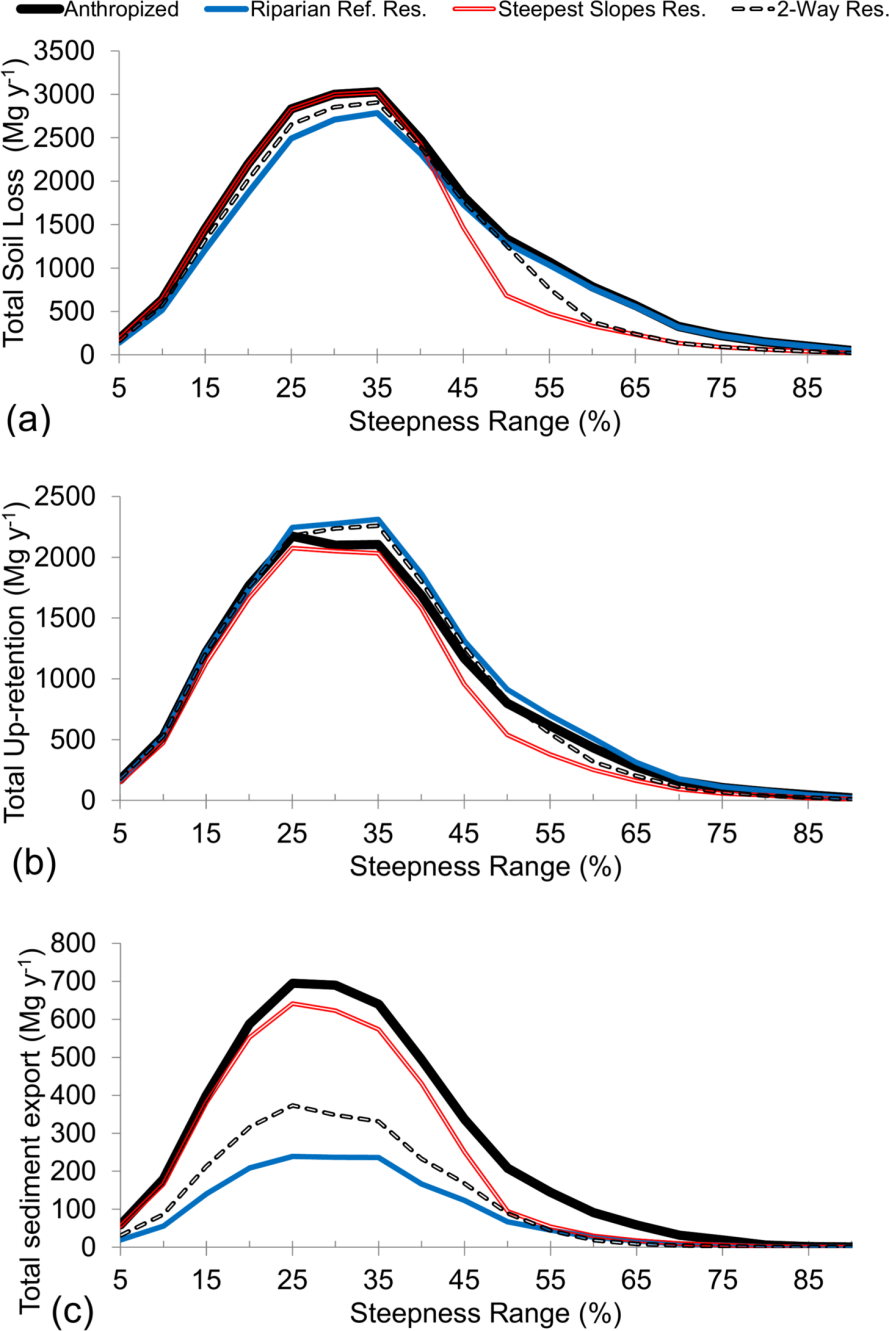
Total soil loss (a), in Mg ha^-1^, total Upstream Sediment Retention (abbreviated as Up-Retention) (b), in Mg ha^-1^, and total sediment export (c), in Mg y^-1^, as function of steepness range, for the Anthropized Scenario and different restoration strategies, with the same area of forest (9% of the area with forest): the steepest slope restoration in areas with declivity greater than > 43%, riparian restoration of 20 m, and 2-Way restoration of 10 m & > 52%.

To analyze and compare the effect of the different restoration strategies across the watershed, one scenario of each restoration strategy (Riparian, the Steepest Slopes and 2-Way, both with the same area of forest as that of the scenario of riparian restoration of 20 m) were compared to the Anthropized Scenario (with no forest). Fig 11 shows total soil loss, upstream sediment retention, and sediment export as function of steepness range (slope). Soil loss increases with slope, but it also depends on the area of the watershed for each steepness range, and this is the reason why it reaches a maximum between 15 and 50% of slope (Fig 11A). Total soil loss was consistently greater or equal in the Anthropized Scenario in all the steepness ranges. For the steepness of 43%, total soil loss started to decrease in the steepest slope restoration in relation to the Anthropized Scenario (this was expected because this scenario considered the restoration for areas with slope above 43%). The Riparian Restoration Scenario produces less soil loss than the Anthropized Scenario between the steepness ranges from 15% to 40% mainly. The 2-Way strategy consistently decreased soil loss in all the steepness ranges, acting as an intermediate between riparian and the steepest slopes restoration.

Restoration decreased Upstream Sediment Retention for the steepest slopes strategy and increased it for riparian and 2-Way restoration (Fig 11B). This non-linearity is due to its dependency on both total soil loss and the efficiency of sediment retention of sediments originating upstream of each pixel. Riparian vegetation retains soil eroded upstream, and so Upstream Sediment Retention was higher in the strategy as compared to the Steepest Slopes Restoration strategy. This strategy, in turn, is more effective in protecting areas susceptible to erosion than retaining the eroded soil, thereby reducing Upstream Sediment Retention.

Sediment exported (Fig 11C) depends not only on soil loss but also on upstream sediment retention. Riparian restoration presents the greatest decrease in sediment exported especially because of the increase in upstream sediment retention (Fig 11B). For the steepest slopes restoration, the decrease is comparable to riparian restoration only for steepness range greater than 43%, where the decrease in soil loss is higher.

**Fig 12:**
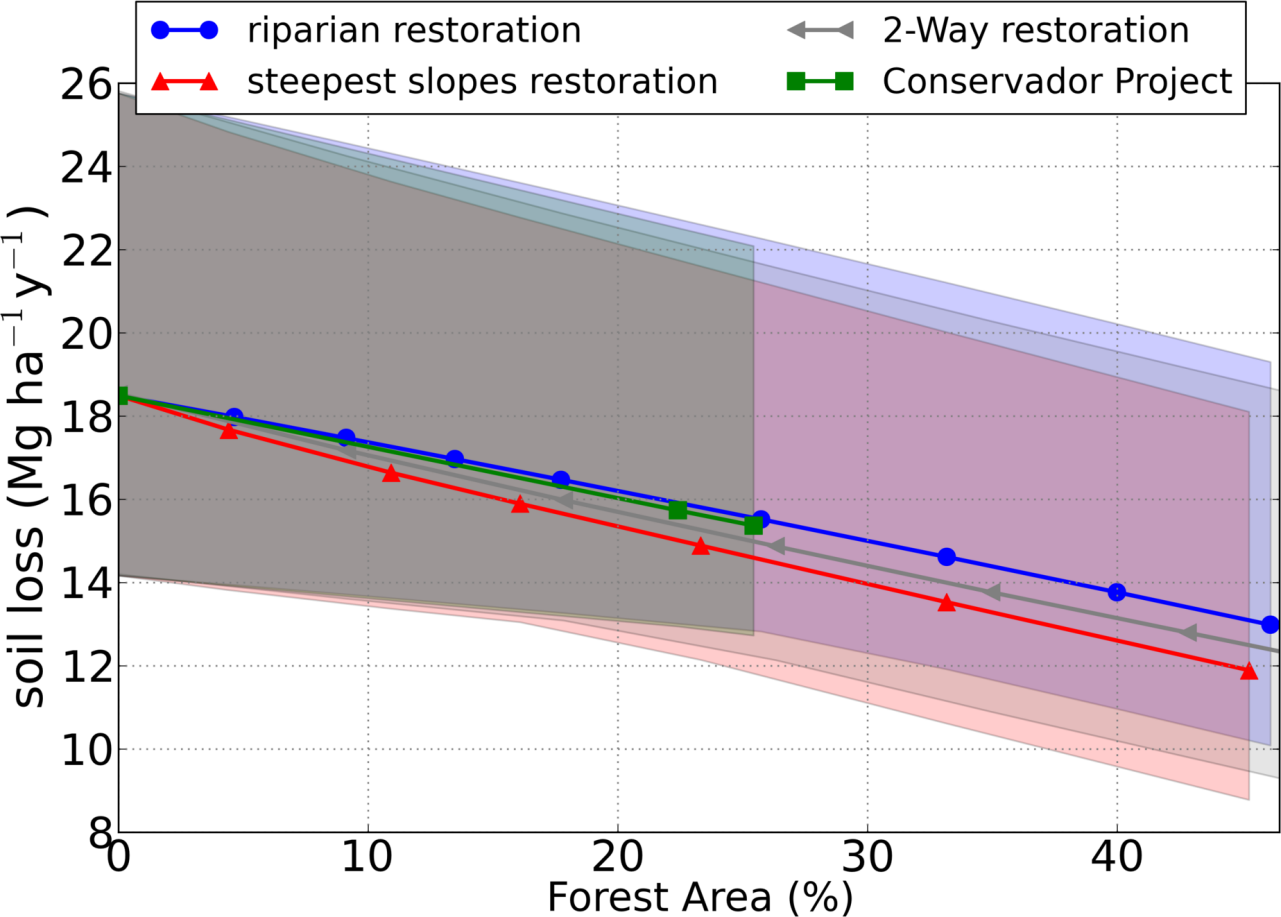
Soil loss simulations, in Mg ha^-1^y^-1^, as a function of forest area for scenarios with different reforestation strategies: Riparian Reforestation (in blue), the Steepest Slopes Reforestation (red), 2-Way restoration (gray), and the Conservador das Águas Project (green). The shaded areas represent the range between the calibration members, and the lines represent the mean of the calibration members. Each geometric symbol in the graph is a restoration scenario (see Fig 9G for more details).

Fig 12 shows the decrease in soil loss with increasing forest area, which was more pronounced for the Steep Slopes Restoration, followed by the 2-Way restoration, Conservador das Águas Project restoration, and the Riparian restoration. The steepest slopes restoration was the strategy which most reduced soil loss due to the effect of increasing soil loss with increasing slope, which was predominant over other landscape characteristics as, for example, soil type (not shown).

**Fig 13:**
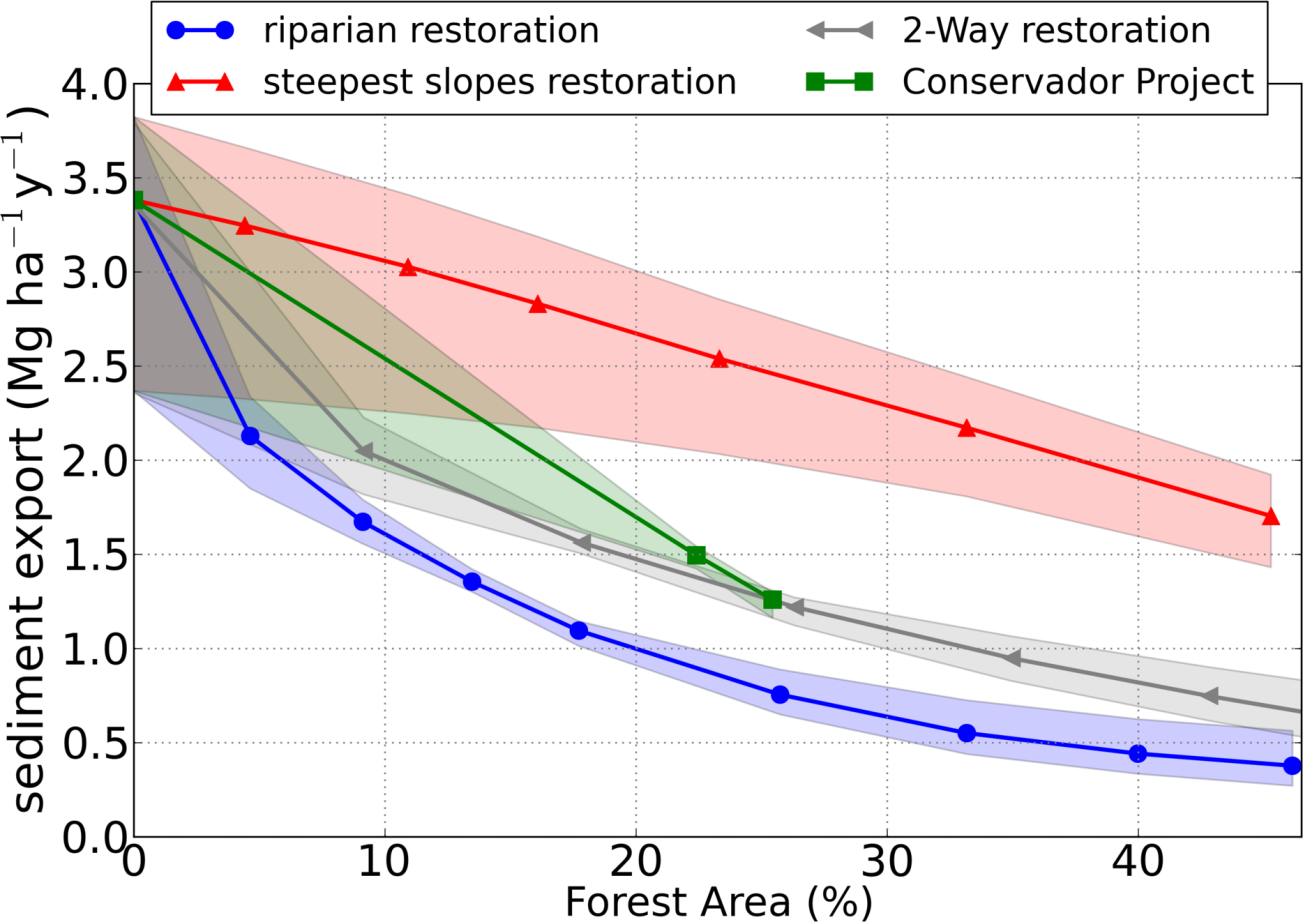
Sediment export simulations, in Mg ha^-1^y^-1^, as a function of forest area for scenarios with different restoration strategies: Riparian Restoration (in blue), the Steepest Slopes Restoration (red), 2-Way restoration (gray), and the Conservador das Águas Project (green). The shaded areas represent the range between the calibration members, and the lines represent the mean of the calibration members. Each geometric symbol in the graph is a restoration scenario (see Fig 9G for more details).

Sediment export (Fig 13) decreased with forest area much more than soil loss did (Fig 12), and the differences in the restoration strategies were much more evident: the Riparian restoration strategy was the one that most decreased sediment export, followed by 2-Way, Conservador Project, and Steepest Slopes. Moreover, this order of efficiency order was practically the opposite of soil loss, despite the fact that sediment export increases with soil loss. This may be explained by the fact that the role of forests could be less related to avoiding soil loss inside a grid cell of the model (illustrated by soil loss reduction in Fig 11A) but will trap the upstream sediments that reach it (illustrated by upstream sediment retention in Fig 11B), behaving as a vegetation filter strip, as described by many previous studies [42,44,49,50]. Thus, the riparian restoration, mostly in the lower parts of watershed, can trap sediments coming from all parts of the watershed and protect the rivers.

The range between the members is given in the shaded areas in the graphs (Fig 12 and Fig 13), and it represents the uncertainty across the scenarios due to parameter choice and input data of this modeling experiment. The soil loss range was high for all percentages of forest area, and especially for 0% of forest, where soil loss is greater. The reason for this high variation is that the calibration was not performed in terms of soil loss (only sediment export) due to the inexistence of soil loss observed data, and the difference between the members was high even in the calibration step (as seen in Table 6). The sediment export range (Fig 13), is much lower than that from soil loss. The sediment export ranges were consistently higher in the Anthropogenic Scenario (0% of forest), where sediment export is higher and where forest area is more different from the Current Scenario, used for calibration.

**Fig 14:**
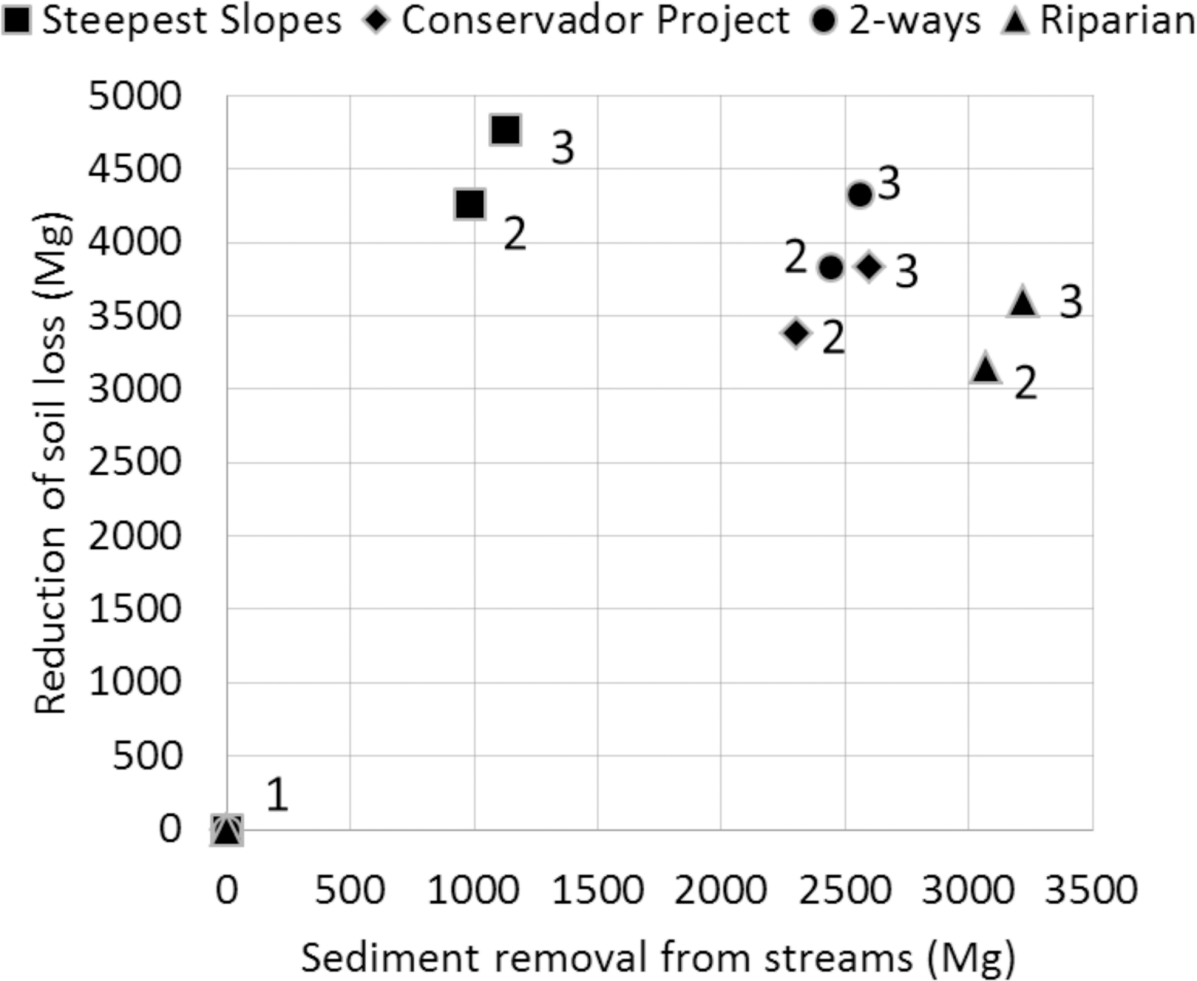
Comparisons between the reduction in soil loss and sediment removal from streams (both in Mg), achieved for the scenarios of 0% (symbols with number 1), 22% (2), and 25% of forest (3) for the different restoration strategies: Conservador Project, Riparian, Steepest slopes, and 2-way. Based on Barnett et al. [9] analysis for evaluation of the delivery of multiple ES.

Fig 14 sums up the effect of the different restoration strategies on sediment transport. The further the points (scenarios) are to abscissa and ordinate axes, the better they are at delivering the ES of reduction of soil loss and sediment export, respectively. All restoration strategies improved both indicators: the Riparian restoration strategy favored sediment export reduction; and the Steepest Slopes Restoration favored the decrease in soil loss. The Conservador Project, and especially 2-Way strategies, favored both services with an intermediate intensity.

## Discussions and limitations

Calibration was performed in terms of sediment export, but not of soil loss, due to the lack of soil loss monitoring in the Posses watershed so far, which could be achieved by plot studies (which is beyond the scope of this research). To work around this limitation, we found not one but several possibilities of soil loss for current land use, due to different calibration combinations. And this uncertainty is also reflected in the other land use scenarios evaluated. For the current land-use scenario, our soil loss estimate was between 12.8 and 22.3 Mg ha^-1^y^-1^ with a sediment export rate of 1.4 Mg ha^-1^y^-1^. The difference in soil loss between the land use scenarios was small in comparison to the estimated uncertainties in each scenario. This was not the case of sediment export, for which the prediction for each restoration strategy was very distinct from the others. However, there might be other uncertainties that were not considered in this study, like the “observed” sediment export, which in an ideal situation would require more measures especially under higher discharge events, as well as direct measures of sediment export, and not only turbidity. Studies using model comparisons would also be advisable for a better estimation of uncertainties as we estimated only the uncertainties related to parameter choice and input data using the InVEST model.

The Brazilian Forest Act requires that an area with 30 meters on both sides of rivers must be covered by vegetation, although the width of restoration buffer depends on the width of the river and the size of the property. We found that sediment export would be reduced in a situation with 30 meters of vegetation strip compared to that without riparian forests from 3.4 to 0.8 Mg ha^-1^y^-1^, in the Posses watershed, an 81% decrease.

The effect of land-use improvement can be measured in two ways: by a comparison with the initial conditions of land use, and by a comparison with a scenario with no conservation management, where all the remnant forest was converted to pastureland, a pessimistic scenario considering the absence of any protection legislation (as Forest Act) or conservation/restoration projects. In this context, the PES system employed in the watershed is important to increase the vegetation coverage with native species, and, more importantly, to maintain remnants. In terms of the ES evaluated, the Post-Project scenario was responsible for decreasing sediment export by 16% in relation to the Pre-Project scenario, and by 63% in relation to an Anthropized Scenario, with no conservation management, with all the remnant forest converted to pastureland. Soil loss decreased only 2% in comparison to the Pre-Project, while it decreased by 17% when compared to the Anthropized Scenario (Table 7).

**Table 7:** Soil loss and sediment export reduction in Post-project in relation to scenarios to Pre-Project and Anthropized.

	Post-project reduction in relation to	
	Pre-project	Anthropized
Soil loss	2%	17%
Sediment export	16%	63%

We showed that riparian restoration was much more efficient in decreasing sediment export than the steepest slope restoration. For example, with 25% of forest, it decreased 78% in comparison to a scenario with no forest, while the steepest slope restoration decreased it only by 27%. We explained this difference mainly to the change in sediment retention, which increased (by 1%) for the riparian restoration and decreased (by 15%) for the steepest slopes. Sediment retention increased for the riparian restoration because the forest buffer had trapped more sediments, and it decreased for the steepest slope restoration because of the decrease in soil loss. It is worth noting that one issue that should be taken into account in further studies is how far the vegetation buffer can increase sediment retention, as occurred in the riparian restoration strategy. Would there be a saturation of this trapping capacity (for example, how long after a precipitation event and with which intensity)? As this possibility of saturation was not considered, there is a possibility that the decrease shown in the sediment export due to the riparian restoration is overestimated, and, as a precaution, we recommend that the steep slope restoration should also be considered in actions intended to protect the quality of the rivers, even because this restoration strategy obtained greater reduction in the soil loss. Moreover, this study did not consider the landslip risk as InVEST does not simulate this process but only the long-term average of soil loss. But as they are related [51], it is expected that the landslip risk may also decrease with restoration, and mainly for the steepest slopes restoration, the strategy which has shown the largest decrease in soil loss.

The effect of roads on general sediment transport is a complex issue that could be investigated in further studies. Here roads accounted for 12% of total sediment loss in the watershed, and this effect could be even higher if the real conditions of the roads were considered, as in the absence of road conservation practices, they increase sediment connectivity. One of the main actions of Conservador das Águas project was to improve road conditions, and we may have overestimated the soil loss in the Conservador das Águas Project scenario as we did not considered road improvements.

## Conclusions

Under different restoration strategies, we evaluated changes in two components of sediment transport across the Posses watershed: soil loss and sediment export. The simulated soil loss in each part of the watershed is routed downslope and downstream, until reaching the water bodies (which characterizes sediment export). Part of the sediment is trapped by vegetation before it reaches streams and reservoirs. We suggested that the decrease in sediment export is controlled not only by the decrease in soil loss, but mostly by the efficiency with which each land use type traps sediments. This is clear when comparing two opposite restoration strategies: one which prioritizes riparian zones, and one that prioritizes the steepest slopes. The Steepest Slopes restoration was the strategy that promoted the greater decrease in soil loss, due to the higher soil loss on the steepest slopes, and Riparian restoration promoted the greater decrease in sediment export, due to the proximity of the vegetation buffer to the rivers, attenuating sediment transported even from the upper parts of the watershed. In the scenarios with an area of 25% of forest (the area achieved with the PES project) soil loss decreased from 18.5 Mg ha^-1^y^-1^ in the Anthropized Scenario (no forest) to 14.6 (21% decrease) and to 15.6 Mg ha^-1^y^-1^ (16% decrease) for restoration of steepest area and riparian, respectively. Sediment export decreased from 3.4 Mg ha^-1^y^-1^ in the Anthropized Scenario to 2.5 (27% decrease) and 0.7 Mg ha^-1^y^-1^ (78% decrease) for Restoration of the Steepest Area and Riparian Restoration, respectively. Despite the apparent superiority of riparian restoration in comparison to the steepest slope restoration in actions to protect river water quality and avoid silting, we highlight the fact that forests can have a limited capacity of trapping, due to the possibility of trapping saturation over time during rainfall events. Thus, the steepest slopes, with higher erosion rates, should also be treated as priorities to avoid the arrival of sediments in rivers.

These processes of vegetation decreasing soil loss and sediment export can be considered as Ecosystem Services (ES), and both are important. The first is related to soil stability, which consequently decreases risks of landslip, and to maintain agriculture productivity. The second may be seen as an improvement in the water quality, which also decreases the risks of silting, with positive effects on the downstream water reservoir. Thus, the Riparian and Steepest Slopes restoration strategies are complementary in the sense of preventing sediments from reaching the water bodies as well as protecting it in its origin (with the reduction of erosion), so it is necessary to consider the two types of restoration to achieve an optimal result. A scenario that considered the restoration performed in the riparian zones and on the steepest slopes simultaneously (2-Way strategy), delivered these two services with intermediate intensity in comparison to the other two strategies. The actions adopted by Conservador das Águas Project resulted in a similar pattern, except that the reduction in soil loss was not as efficient as in 2-Way experiment. However, other services are also needed to be evaluated, like for example the water quantity in the springs, which was one of the priorities of Conservador das Águas Project.

Further works could also consider other restoration strategies, like restoring vegetation around springs, and the effects of restoration on low and extreme flows, biodiversity, and carbon emission. These Ecosystem Services may have been strengthened by the Conservador das Águas Project, whose interventions may have also benefited the environmental and social conditions of the small land owners inhabiting the watershed.
